# A strategy for prevention and control of catheter-related bloodstream infection of ICU patients in China (Prevent CRBSI): a prospective, multicenter, controlled study

**DOI:** 10.1186/cc10682

**Published:** 2012-03-20

**Authors:** G Cai, J Yan

**Affiliations:** 1 Zhejiang Hospital, Hangzhou, China

## Introduction

Catheter-related bloodstream infection (CRBSI) continues to be a key issue in ICUs despite recent improvements in the clinical technique, standardization of the CVC insertion protocol and hand hygiene. The impact of catheter maintenance on CRBSI rates in China needs to be further investigated. The objective of study is to evaluate a bundle of interventions for reducing CRBSI in ICUs. The bundle includes new technology (BD Q-Syte™ and BD Posiflush™) in addition to updated standards of practice for catheter maintenance.

## Methods

This is a prospective, multicenter, controlled study. Patients receiving CVCs in the ICUs were eligible for inclusion. The study was performed in seven general and teaching hospitals from June 2010 to June 2011 in China. The clinicians conducted their original catheter maintenance standards in the baseline period (Phase 1). The bundle was introduced in Phase 2. CRBSI was determined according to US CDC diagnostic criteria. The rates of CRBSI before and after the introduction of the bundle of interventions were compared.

## Results

A total of 619 patients were enrolled in the study. During the baseline period (Phase 1), 238 patients with 2,456 catheter days were assessed, 30 patients developed a CRBSI. The CRBSI rate during this period was 12.2 per 1,000 catheter days. All nurses and principle doctors in the seven ICUs received training on the standard of care for catheter maintenance along with the introduction of Q-Syte™ and Posiflush™. In Phase 2, following introduction of the interventions, 12 of 381 patients developed a CRBSI. Total catheter days during this period were 3,562. The CRBSI rate decreased to 3.4 per 1,000 catheter days. This was significantly lower than during the baseline period (Wilcoxon nonparametric test, *u *= 4.36, *P = *0.0003). Additional analyses demonstrated that patients were at higher risk for developing a CRBSI if associated with: a prolonged catheter dwell time, a higher number of insertion attempts, a blood infusion or an increased frequency of catheter connector changes. We also found that the patients who developed a CRBSI had prolonged hospital stay and significantly added to the cost of treatment.

## Conclusion

Introduction of Q-Syte™ and Posiflush™ and improved standards of practice for catheter maintenance can significantly decrease CRBSI in the ICU.
